# Supplementary material for: Progressive Platelet Rich Fibrin tissue regeneration matrix: Description of a novel, low cost and effective method for the treatment of chronic diabetic ulcers—Pilot study
Source: PLoS One. 2023 May 4;18(5):e0284701. doi: 10.1371/journal.pone.0284701 (PMC10159142; doi:10.1371/journal.pone.0284701)
Supplement: S1 File — (PDF) [file pone.0284701.s001.pdf]

APR 13, 2023

Wound Care Protocol (step-by-step protocol)

rodrigo.raimundo[1]

[1]Faculdade de Medicina do ABC

rodrigo.raimundo

ABSTRACT

Chronic lower limb ulcers (CLLU) are those injuries that persist for more than six weeks despite adequate care. They are relatively common; it is estimated that 10/1,000 people will develop CLLU in their lifetime. Diabetic ulcer, because of its unique pathophysiology (association between neuropathy, microangiopathy, and immune deficiency), is considered one of the most complex and difficult etiologies of CLLU for treatment. This treatment is complex, costly, and sometimes frustrating, as it is often ineffective, which worsens the quality of life of patients and makes its treatment a challenge

OPEN ACCESS

DOI: dx.doi.org/10.17504/protocols.io.n2bvj8wqpgk5/v1

Protocol Citation: rodrigo.raimundo 2023. Wound Care Protocol (step-by-step protocol). **protocols.io** https://dx.doi.org/10.17504/protocols.io.n2bvj8wqpgk5/v1

License: This is an open access protocol distributed under the terms of the Creative Commons Attribution License, which permits unrestricted use, distribution, and reproduction in any medium, provided the original author and source are credited

Protocol status: Working
We use this protocol and it's working

Created: Apr 12, 2023

Last Modified: Apr 13, 2023

PROTOCOL integer ID: 80387

Wound preparation

1 Cleaning and antisepsis of chronic wounds were performed with an aqueous solution of chlorhexidine and 0.9% saline before applying the blood concentrates

Blood Concentrate Preparation (PRO-PRF)

2 Blood samples were obtained by venipuncture, the patient remained comfortably seated, the upper limb was tourniqueted with an elastic band (similar to the usual collection of blood samples), using collection needles (scalp) and plastic tubes (PET), without blood collection additives (VACUETTE®, Grainer, Americana, São Paulo, Brazil).

3 The collection of blood samples must be completed within 05 minutes, as exceeding this time, the blood clotting process can alter the quality of the samples.

4 Ten to 12 tubes were collected, depending on the extent of the wound and the time of collection, and immediately transferred to a fixed-angle rotor centrifuge (DT 4000 Daiki Lab Bran Digital Centrifuge), with a total capacity of 12 tubes, and centrifuged to produce o PRO- PRF Blood Concentrate

5 We used a new progressive centrifugation protocol, developed by Saboia-Dantas and Dechichi (10). Blood samples were centrifuged for a total time of 15 minutes, with progressive and sequential increase in speed (RPM - rotations per minute) at intervals of 05 min (700 RPM / 1300 RPM / 2400 RPM)

6 After centrifugation, the blood concentration obtained in each tube was aspirated with a 10 ml hypodermic syringe and a 16G needle. A small fraction of this material (PRO-PRF in the fluid phase) was immediately inoculated into the margins and perimeter of the lesion.

7 The largest fraction of the PRO-PRF obtained was deposited in a receptacle (Petri dish) for the modeling of a PRO-PRF concentrate, remaining for an average period of 15 minutes (sufficient time for the sample to acquire a membranous consistency), during this period, the membrane being formed is manipulated and rotated 180 degrees in the container so that both sides of the membrane come into contact with the ambient air, homogenizing the preparation

8 Next, the excess water is removed through gentle pressing, carried out in a stainless-steel box with porosities for liquid drainage and a 30-gram glass plate (PRF Box) for draining the whey. In this way, the Giant PRO PRF membrane (GMPro) is obtained. The liquid resulting from this process was aspirated and inoculated into the wound bed

PRO-PRF application and wound protection

9 The PRO-PRF, still in the fluid phase, was immediately inoculated onto the edges of the CLLU and deposited on the wound surface. In the next step, the PRO-PRF membrane was implanted in the wound bed and fixed and stabilized with cyanoacrylate adhesive glue

10 Finally, the wound was covered and wrapped in a film dressing made of polyvinyl chloride (PVC), porous multiples of approximately 0.5 cm 2 were made around the entire perimeter of the wound. These pores were made for the drainage of the transudate that forms during the biodegradation of the membrane. Secondarily, gauze was placed to absorb this transudate and bandages
